# Targeted Drug Delivery Systems for Kidney Diseases

**DOI:** 10.3389/fbioe.2021.683247

**Published:** 2021-05-28

**Authors:** Xiaohan Huang, Yanhong Ma, Yangyang Li, Fei Han, Weiqiang Lin

**Affiliations:** ^1^Key Laboratory of Kidney Disease Prevention and Control Technology, Kidney Disease Center, Zhejiang University School of Medicine, The First Affiliated Hospital, Institute of Nephrology, Zhejiang University, Hangzhou, China; ^2^Key Laboratory of Women’s Reproductive Health Research of Zhejiang Province, Women’s Hospital, Zhejiang University School of Medicine, Hangzhou, China; ^3^Department of Nephrology, The Fourth Affiliated Hospital, Institute of Translational Medicine, Zhejiang University School of Medicine, Jinhua, China

**Keywords:** kidney diseases, nanomaterial, targeted delivery, renal tubules, glomeruli

## Abstract

Kidney diseases have gradually become a global health burden. Along with the development of nanotechnology, many hybrids or nanomaterials have been utilized to promote treatment efficiency with negligible side effects. These therapeutic agents have been successfully applied in many fields. In particular, some efforts have also been made to ameliorate the treatment of kidney diseases through targeted delivery nanomaterials. Though most of the delivery systems have not yet been transmitted into clinical use or even still at an early stage, they have shown great potential in carrying immunosuppressants like tacrolimus and triptolide, antioxidants, or siRNAs. Excitingly, some of them have achieved significant treatment effectiveness and reduced systemic side effect in kidney disease animal models. Here, we have reviewed the recent advances and presented nanotherapeutic devices designed for kidney targeted delivery.

## Introduction

The kidney is a vital organ that filters blood and removes excess fluid and waste products. Dysfunction of the kidney may break the fluid and electrolyte balance, and lead to severe problems. However, it can happen undetected until it progressed into advanced stages, so kidney diseases are often considered a silent killer. Common kidney diseases includes IgA nephropathy, membranous nephropathy, acute kidney injury etc. And kidney impairment may also happens secondary to other diseases including diabetes, hypertension and autoimmune diseases. It has been reported that the incidence of chronic kidney disease is reported to be around 11% (Webster et al., 2017). And according to the US Centers for Disease Control, about 37 million US citizens are estimated to have chronic kidney disease, and nearly 0.7 million are in end-stage kidney disease, needing life-sustaining dialysis or kidney transplant (Centers for Disease Control and Prevention, 2021. Chronic Kidney Disease Surveillance System^1^; Accessed January 7). However, the treatments for kidney diseases are limited. Commonly used medications for kidney diseases including steroids and immunosuppressants tend to have systemic toxicity. Therefore, it is important to improve the safety and efficiency of treatment.

With the development of nanotechnology, a variety of hybrids, including nanoparticle-platform-based therapeutic agents, have emerged. Medical investigators have gradually realized that nanotechnology might contribute potential advances to basic medical research and clinical practice (Zhou et al., 2014; Kamaly et al., 2016; Liu et al., 2019). The nanoparticle-based drug delivery platform enables therapeutic agents can specifically accumulate at diseased sites (Blanco et al., 2015), and thus reduce undesired systemic effects. Also, targeting specific components of the kidney may provide new approaches to refractory diseases. For example, targeting tubular cells to reduce fibrosis, and targeting mesangial cells to ameliorate mesangial proliferative glomerulonephritis.

Here we present recent developments of nanoparticles designed for kidney targeted delivery and briefly introduce targeting strategies for the kidney (Table 1).

**TABLE 1:** Nanomaterials for kidney targeted drug delivery.

Target	Material	Particles	References
Tubules	PLGA	Mesoscale nanoparticles (MNPs) PLGA-PEG	Williams et al., 2015, 2018; Deng et al., 2019; He et al., 2020
Tubules	PLGA, ceria	Ceria nanoparticles modified with triphenylphosphine (TCeria NPs) and coated with ROS-responsive organic polymer	Yu et al., 2020
Tubules	Chitosan	Chitosan/siRNA nanoparticles mediated by megalin dependent endocytic pathway	Gao et al., 2014; Yang et al., 2015
Tubules	Chitosan	Catechol-derived low molecular weight chitosan	Luo et al., 2019
Tubules	Chitosan	Catechol-derived low molecular weight chitosan	Qiao et al., 2014; Li et al., 2017
Tubules	Chitosan, PLGA	MicroRNA inhibitor- LMWC-modified PLGA nanoparticles	Geng et al., 2018
Tubules	Chitosan	l-serine–modified chitosan -TK-SS31	Liu et al., 2020
Tubules	PAMAM	PAMAM dendrimer-based macromolecular conjugate of a multitargeted sunitinib analog	Dolman et al., 2012b
Tubules	PAMAM	l-Serine modified PAMAM dendrimers	Matsuura et al., 2018a, b; Katsumi et al., 2019
Tubules	pSi	pSi NP displaying with monoclonal antibodies loaded with rapamycin	Stead et al., 2018; Zhang et al., 2019
Tubules	Protein	DNA-binding protein from starved cells (Dps)	Uchida et al., 2019
Glomeruli	Liponanoparticles, peptide	Kidney-targeted rhein (RH)-loaded liponanoparticles (KLPPR)	Wang et al., 2019
Glomeruli	Antibody	Human monoclonal anti-α3(IV) antibody	Bruni et al., 2017
Glomeruli	Antibody	Anti-VCAM-1-rapamycin-SAINT-O-Somesthat	Visweswaran et al., 2015
Glomeruli	PHMAM, peptide	PHMAM copolymer integrinαvβ3-specific cyclo-RGD	Pollinger et al., 2012
Glomeruli	Gold	PEGylated gold NPs	Choi et al., 2011
Glomeruli	Antibody, liposomes	Immunoliposomes coated with anti-integrinα8 monoclonal antibodies or anti-Thy-1-membrane glycoprotein (Thy1)	Scindia et al., 2008; Suana et al., 2011
Glomeruli	Peptide, liposomes	PEG-modified liposome with desired size (110 nm) and octa-arginine (R8) coating	Wang et al., 2020

*PLGA, Poly(lactic-co-glycolic acid); PAMAM, Polyamidoamine; pSi, porous silicon; PHMAM, Poly(N-2hydroxypropyl)meth acrylamide; PEG, polyethylene glycol; NP, nanoparticles; CaPi, calcium phosphate; LWMC, low molecular weight chitosan.*

## Targeted Delivery for Renal Tubulointerstitial Diseases

### Targeting Strategies

Renal tubules include the proximal tubule, the loop of Henle, the distal tubule, and the collecting ducts. Renal tubulointerstitial injuries including renal interstitial inflammation or fibrosis and tubular atrophy are the common result of kidney diseases and are crucial in the development of chronic kidney disease and end-stage kidney diseases. They can occur secondary to vascular and glomerular diseases, or primary tubulointerstitial diseases, for example, acute kidney injury (Dolman et al., 2010; Grgic et al., 2012; Falke et al., 2015; Ramos et al., 2015). Overactivated immune system and oxidative stress play important roles in the pathogenesis of the diseases.

Proximal tubule targeted therapeutics including inhibitors of the renin-angiotensin-aldosterone system, steroids, and immunosuppressants can be promising drugs to inhibit tubulointerstitial fibrosis and prevent the progression of chronic renal diseases (Dolman et al., 2010; Falke et al., 2015). And antioxidants targeting renal tubules may protect the tubular cells from ROS- induced oxidative stress (Wang et al., 2021).

**FIGURE 1:**
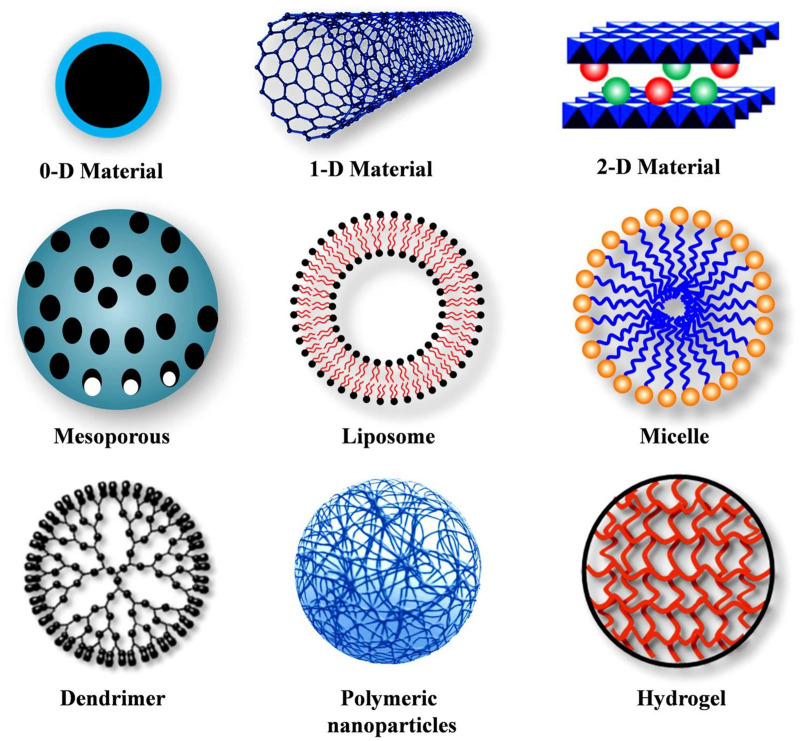
Different types of nanocarriers. Reproduced with permission (Senapati et al., 2018).

Various forms of nanomaterials have been used to design drug carriers (Figure 1; Senapati et al., 2018). To target renal tubules, surface chemistry, size, and charge can be important factors.

#### Size, Charge, and Surface Chemistry

**FIGURE 2:**
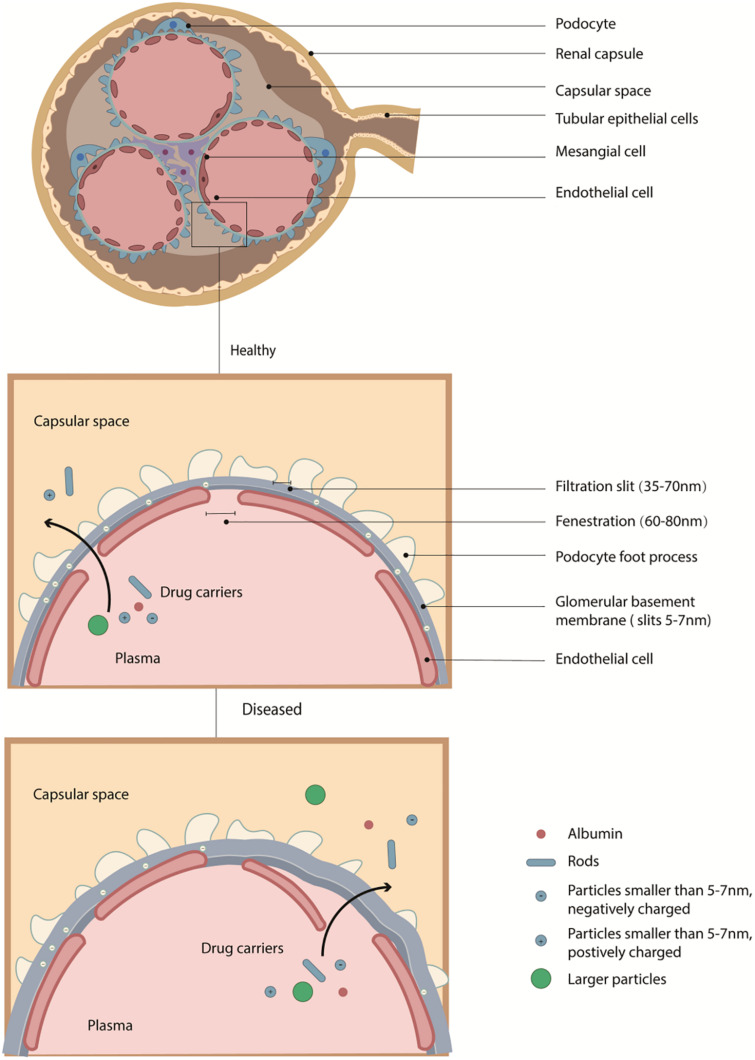
The structure of glomerulus and the glomerular filtration membrane. The fenestration of endothelial cells sized about 100 nm. And the glomerular filtration barrier in a healthy kidney is negatively charged with slits at about 5–7 nm. And the fenestration between the foot process of podocyte is 35–70 nm. In healthy conditions, the filtration membrane can block negatively charged proteins and particles, and large molecules, but in the diseased situation, both the size or charge control of the barrier can be impaired, allowing particles with larger size passing through.

The fenestration of the peritubular capillary is sized 60–70 nm (Dolman et al., 2010). And the glomerular filtration barrier in a healthy kidney is negatively charged with slits at about 6–10 nm (Oroojalian et al., 2020). The barrier allows small molecules with diameters less than 6 nm (including NPs with high aspect ratios but small diameters, for example, rods, tubes, and sheets) to pass through (Figure 2; Park et al., 2008; Chauhan et al., 2011; Jasim et al., 2016). Of note, though some small NPs sized less than 10 nm or proteins smaller than 20 kDa can distribute at tubules, they may not be able to retain in the body (Dolman et al., 2010).

Due to charge selectivity (Comper and Glasgow, 1995), NPs with negative surface charges are more likely to be blocked out by the filtration barrier (Choi et al., 2009). But interestingly, some studies found that strongly negatively charged NPs may pass the barrier faster than weakly negatively charged or positively charged NPs in some situations, and thus further investigation should be provided to prove detailed discussion (Kang et al., 2016; Yu et al., 2017). High dose of NPs with positive surface charge may have cytotoxicity due to disturbtion to resting potential.

Surface modification may affect organ distribution (Amin et al., 2015; Kokkinopoulou et al., 2017). Before reaching kidney tissues, NPs should try to reduce the chance of absorbing serum proteins which may form protein corona (Salvati et al., 2013; Jiang et al., 2018; Richtering et al., 2020) and avoid being captured by the reticuloendothelial system of the liver (slits about 50–100 nm), the spleen (slits about 200–500 nm), or other organs (Klibanov et al., 1990; Du et al., 2017; Sun et al., 2017). It has been suggested that surface modifications like polyethylene glycol (PEG) molecules can reduce serum protein adsorption, and thus avoid being captured by the reticuloendothelial system (Harris and Chess, 2003; He et al., 2011). Meanwhile, the negatively charged surface will help to decline the formation of protein corona *in vivo* (Alexis et al., 2008; Blanco et al., 2015).

In the diseased situation, both the size and surface charge control of the barrier can be impaired (Pavenstädt et al., 2003; Du et al., 2018), allowing particles with larger sizes to pass through. Still, it should avoid being captured by the liver, spleen, or other organs. Of note, in kidney diseases, inflammation usually leads to acidosis at diseased sites, and thus PH triggered systems can be used to design targeted delivery systems.

#### Molecular Recognition and Endocytosis

**FIGURE 3:**
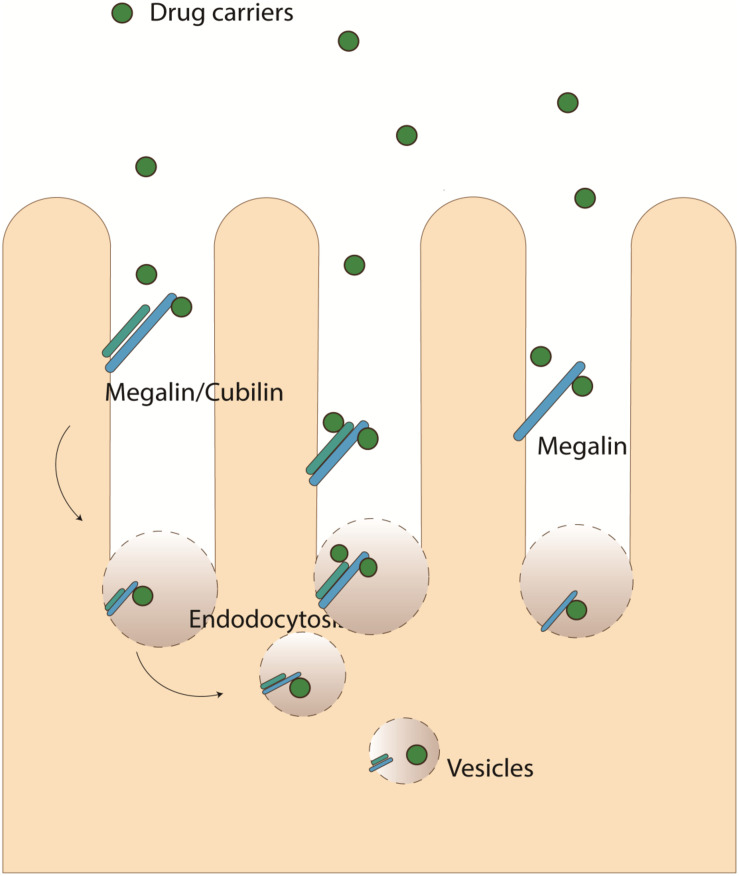
Megalin/cubilin mediated endocytosis.

Molecular recognition moiety, including antibody, small molecule, or aptamer, can be used as another strategy. These moieties can be recognized by certain surface receptors and thus binds to renal cells. For example, drugs containing certain surface molecules can achieve kidney-targeted delivery through Megalin/cubilin-mediated endocytosis (Figure 3) by the luminal membrane of tubular epithelial cells (Williams et al., 2016). Megalin is a low-density lipoprotein (LDL) receptor that is expressed in epithelial cells of the intestine, kidney, and other tissues (Christensen and Birn, 2002; Nielsen et al., 2016). On the apical plasma membrane of proximal tubule cells, it binds with cubilin (intestinal intrinsic factor-cobalamin receptor) and can mediate the endocytosis of many ligands including lipoproteins, hormones, enzymes, lysozyme, carrier proteins, drugs (such as aminoglycoside and polymyxin B and chitosan) (Christensen and Birn, 2002; Lin et al., 2013; Oroojalian et al., 2017; Xu et al., 2020). It is one of the most important pathways for the absorption of many molecules and tubular targeting NPs in renal tubular cells. (KKEEE)_3_K(Lys–Lys–Glu–Glu–Glu]3–Lys) is one of the well-studied peptide ligands that can achieve targeted kidney delivery through megalin-mediated endocytosis of proximal tubule cells (Wang et al., 2018). Variations of (KKEEE)3K peptides possess targeting performance for the kidney (Janzer et al., 2016; Wischnjow et al., 2016; Huang et al., 2020).

### Drug Delivery Systems for Renal Tubules

#### PLGA-PEG NPs

Poly(lactic-co-glycolic acid) (PLGA) conjugated to polyethylene glycol (PEG) is one of the most commonly used polymers to synthesize polymeric NPs (Danhier et al., 2012). The Biosafety of PLGA-PEG has been well-tested and its application in humans has been approved by the FDA. Mesoscale nanoparticle (MNP) is about 400 nm in diameter and is a drug delivery system formed from PLGA-PEG (Williams et al., 2015). By intravenous administration, it can specifically localize at the kidneys (7 times than other organs) and retain in renal epithelial cells for weeks (Williams et al., 2015). The particles can be released to the tubulointerstitium after being endocytosed by peritubular endothelial cells and then uptake by tubular epithelial cells. The capacity of kidney-specific accumulation is dependent on the size and hydrophilic PEG surface while the surface charge does not affect its targeting effect (Williams et al., 2015). The long PEG chains can reduce opsonization and thus avoid been captured by the mononuclear phagocyte system. Mice treated with MNPs displayed primarily tubular localization without obvious side effects (Williams et al., 2018). For the treatment of renal diseases, a research group has successfully constructed triptolide-encapsulated MNPs (TP-MNP). Triptolide is an immunosuppressant and the TP-MNPs showed kidney-specific accumulation and high treatment efficiency in the ischemia-reperfusion AKI animal model (Deng et al., 2019).

PLGA-PEG nanoparticles modified by kidney targeting proteins (KTP) can achieve 30-fold enhanced targeted delivery of Asiatic acid (an anti-inflammatory drug) to renal tubules, and can potentially be used in the treatment of chronic kidney diseases (He et al., 2020). However, this NP carreier may also slightly enhance drug distribution in heart.

#### Chitosan-Based NPs

**FIGURE 4:**
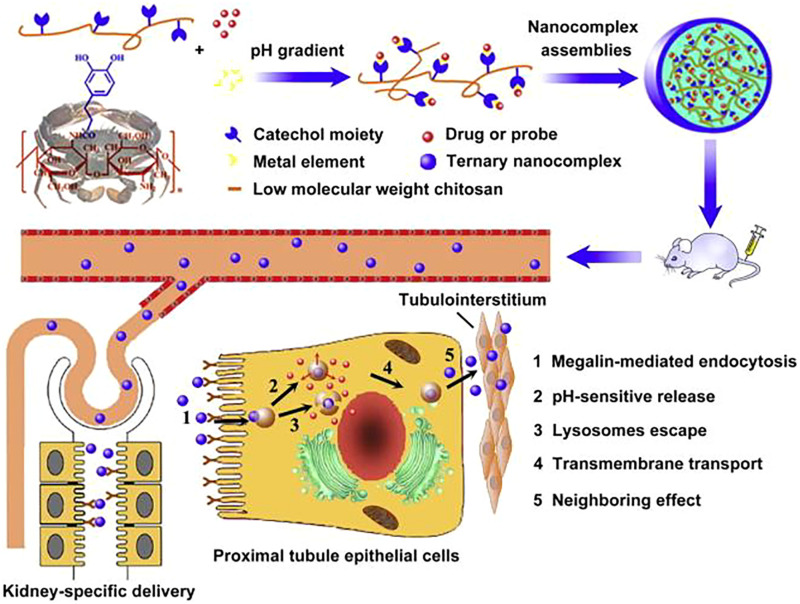
HCA-Chi for kidepH-response nanodevice based on a coordination-driven assembly of HCA-Chi for sequential and kidney-specific drug delivery. Reprinted with permission (Qiao et al., 2014).

Chitosan is another widely used NP material for its great biocompatibility and biodegradability (Narayanan et al., 2014; Kravanja et al., 2019). Chitosan/siRNA nanoparticles also can target siRNA at the tubular cells in mice (Gao et al., 2014; Yang et al., 2015). The uptake was mediated by a megalin-dependent endocytic pathway (Gao et al., 2014). Low molecular weight chitosan-based NPs can increase the uptake by renal tubular cells (Luo et al., 2019). Qiao et al. (2014) designed an NP based on hydrocaffeic acid-containing catechol-derived low molecular weight chitosan (HCA-Chi) and metal ions also showed special renal targeting capacity (Figure 4). The distribution of HCA-Chi outside the kidneys are neglitable. And when loaded with emodin (an antifibrosis agent), the NP can inhibit the progression of renal fibrosis in ureter obstructed mice (Qiao et al., 2014). Also, HCA-Chi modified with calcium and salvianolic acid B reversed the TGF-β1-induced epithelial-mesenchymal transition in HK-2 cells (an immortalized proximal tubule epithelial cell line), and *in vivo* imaging showed a kidney-specific biodistribution (Li et al., 2017). Small-sized cationic microRNA inhibitor- LMWC-modified PLGA nanoparticles also possess kidney-targeting capability and high antifibrosis efficiency (Geng et al., 2018). The drug is two times higher in kidney than heart or liver.

**FIGURE 5:**
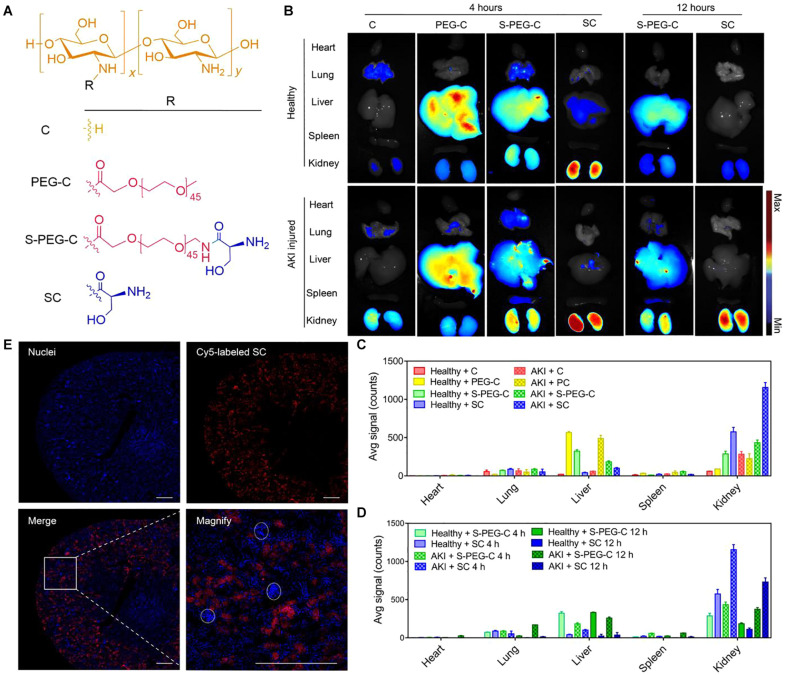
Renal distribution and renal tubules accumulation of l-serine–modified chitosan (SC). Reprinted with permission (Liu et al., 2020). **(A)** The chemical structures of SC and its analogies **(B)** Fluorescence images of the main organs (heart, lung, liver, spleen, and kidney) of mice at 4 or 12 h after intravenous injection of Cy5-C, Cy5-PEG-C, Cy5-S-PEG-C, or Cy5-SC. One of three independent experiments is shown. Renal ischemia was induced by clamping of the bilateral renal pedicles for 30 min and then removed clamping to induce IR AKI. After the initiation of AKI, fluorescence-labeled SC and its analogies were administered intravenously. Four or twelve hours later, the main organs were harvested for fluorescence visualization. The healthy mice were treated with the same protocol as control. **(C,D)** Region of interest analysis of the kidney uptake at 4 and 12 h after injection. The data are the means ± SD. *n* = 3 independent mice. **(E)** Representative confocal images of kidney sections after intravenous injection of Cy5-SC (red signal) for 4 h. Blue indicates 4′,6-diamidino-2-phenylindole (DAPI) staining. White dashed circles denote glomeruli. Scale bars, 500 μm.

A research team from Korea synthesized hydrophobically modified glycol chitosan (HGC) polymeric nanomicelles, and when loaded with tacrolimus, the hybrid showed efficient selective delivery to the kidney and can significantly reduce systemic side effect (Kim et al., 2020). Liu et al. (2020) designed l-serine–modified chitosan-based carrier (SC) and found that it can effectively accumulate at kidney in AKI mice while been cleared by healthy mice (Figure 5). And when ROS-sensitive prodrugs (SS31) were conjugated with the carrier, the drug (SC-TK-SS31) was successfully distributed in tubular cells and showed enhanced protective effect from AKI (Liu et al., 2020).

#### Dendrimers

**FIGURE 6:**
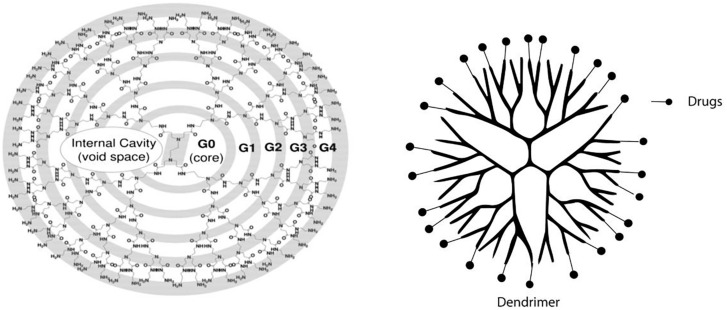
Schematic representation of dendrimer. PAMAM-NH2 G0 to G4 dendrimer starts from an ethylenediamine core; the branches or arms were attached by exhaustive Michael addition to methyl acrylate followed by exhaustive aminolysis of the resulting methyl ester using ethylene diamine. Reprinted with permission (Abbasi et al., 2014).

Polyamidoamine (PAMAM) dendrimer-based NPs are another group of extensively studied drug delivery NPs. Dendrimers are globular macromolecules with highly branched structures that enable efficient conjugation of targeting ligands (Figure 6; Gillies and Frechet, 2005; Abbasi et al., 2014; Huang and Wu, 2018). Several studies demonstrated that I-Serine modified PAMAM (G3) dendrimers are highly potent as a renal proximal tubule- targeted drug carrier (Matsuura et al., 2018a, b; Katsumi et al., 2019) as it accumulates mainly in the kidney and the level in other organs are neglitable. The NPs can be used to prevent ischemia/reperfusion kidney injury as reactive oxygen species (ROS) scavenger. The dendrimers can be filtered by the filtration membrane and be reabsorbed by tubular cells. The mechanism of reabsorption involves caveolae-mediated endocytosis, micropinocytosis, and megalin-mediated endocytosis (Matsuura et al., 2018b). Researchers have found that a high degree of Ser modification (≥80%) was required for effective kidney-specific delivery (Matsuura et al., 2018b). The dendrimer can effectively deliver captopril to the kidney (Matsuura et al., 2018a) and thus reduce its side effects. PAMAM dendrimer-based macromolecular conjugate of a multitargeted sunitinib analog also showed proximal tubular cell-targeted delivery (Dolman et al., 2012b). The sunitinib analog is conjugated to NH(2)-PAMAM-G3 dendrimer with a platinum-based linker and displayed efficient accumulation in rodent kidneys. Besides, the same group conjugate the sunitinib analog to a kidney-specific carrier lysozyme, with antifibrotic effects (Dolman et al., 2012a), suggesting lysozymes can also be used as a targeting ligand (Kamaly et al., 2016).

#### NPs Displaying Antibodies

Porous silicon (pSi) NPs are a nanomaterial with versatile surface modification routes and can be used in controlled drug delivery (Zhang et al., 2019). A pSi NP displaying monoclonal antibodies loaded with rapamycin showed enhanced kidney targeting (Stead et al., 2018). Though originally designed to promote regulatory T cell proliferation, it may serve as a potential treatment for Fanconi syndrome, which is a disease of renal proximal tubules (Stead et al., 2018; Zhang et al., 2019).

#### Other NPs Targeting Renal Tubules

Ultra-small ceria nanoparticles modified with triphenylphosphine (TCeria NPs) and coated with ROS-responsive organic polymer (mPEG-TK-PLGA) can target mitochondria. Kidney and liver are the main accumulation sites in healthy mice and the accumulation in kidney is significantly higher in AKI mice. When loaded with atorvastatin can protect tubular cells from oxidative stress and inflammation in AKI mice models (Yu et al., 2020). However the toxicity of TCeria should be awared.

DNA-binding protein from starved cells (Dps), cage-like protein nanoparticles that are sized less than 9 nm, can pass through the glomerular filtration barrier to be endocytosed by proximal tubules. Its accumulation in other oragans are neglitable comparing to that in the kidney. And based on its characteristics, modified Dps can be a platform targeting renal proximal tubules. Researchers have found that manganese-bound Dps (MnDps) can act as an antioxidant and protect the kidney from endotoxin-induced injury in mice (Uchida et al., 2019).

## Drug Delivery Systems for Glomerular Diseases

### Targeting Strategy for Glomeruli

The glomerulus contains mesangial cells, parietal epithelial cells, visceral epithelium cells (podocyte), glomerular basement membrane (GBM), and glomerular endothelial cells (GEC) (Haraldsson et al., 2008; Jarad and Miner, 2009; Lu and Gu, 2017; Zhao, 2019). And the later three forms the glomerular filtration barrier.

Glomerular diseases are the most common form of kidney diseases. The inflammation of glomeruli is called glomerulonephritis, common forms of glomerulonephritis and cellular location of their injury are listed in Table 2 (Chadban and Atkins, 2005). The pathogenesis of glomerulonephritis usually involves the deposition of abnormal immunocomplexes and overactivation of immune cells. Thus, steroids and immunosuppressants including mycophenolate mofetilare, celastrol, tacrolimus, cyclophosphamide and cyclosporin are the most common therapeutics for glomerular diseases.

**TABLE 2:** Location of injury in glomerulonephritis.

Location	Disease
**Mesangial cell**	IgA nephropathy
	Mesangioproliferative glomerulonephritis
	Class II lupus nephritis
	Diabetic nephropathy
**Endothelial cell**	Infection associated glomerulonephritis
	Membranoproliferative glomerulonephritis
	Class III and IV lupus nephritis
	Anti GBM disease
	Hemolytic uremic syndrome
	Vasculitis
**Epithelial cell**	Membranous nephropathy
	Minimal change nephropathy
	focal segmental glomerulosclerosis
	Class IV lupus nephritis

***Table of Contents:** Kidney disease has become a global health problem. And with nanotechnology, therapeutic agents can achieve selective accumulation at the kidney, and thus reduce the side effect and increase drug concentration on the diseased site. Here we summarized recent advances in nanoparticles designed for kidney targeted delivery according to the targeting sites.*

Currently, limited methods can be used to target glomeruli. Similar to the targeting strategy of renal tubules, size, shape, and especially molecular recognition moiety is important in glomeruli-specific delivery. Of note, diseased glomeruli expose epitopes that are usually sequestered, and thus enhances antibody-mediated targeted delivery. And some surface molecules are upregulated in diseased conditions and thus provide targeting sites for carriers. Renal targeting peptide ligands and antibody ligands have been extensively reviewed by Wang et al. (2017) Here we introduce drug delivery systems to different components of glomeruli.

### Drug Delivery Systems for Renal Glomeruli

#### Drug Delivery Systems for GBM and GEC

GBM is a specialized extracellular matrix between GEC and podocyte, it consists mainly of laminin, type IV collagen, nidogen, heparan sulfate proteoglycan (Miner, 2012). And heparan sulfate proteoglycan contributed to the negative charge of the GBM and thus prevent albumin from being filtered (Kanwar et al., 1980). It is an important barrier limiting the targeted delivery of NPs.

##### NPs Displaying Ligands

**FIGURE 7:**
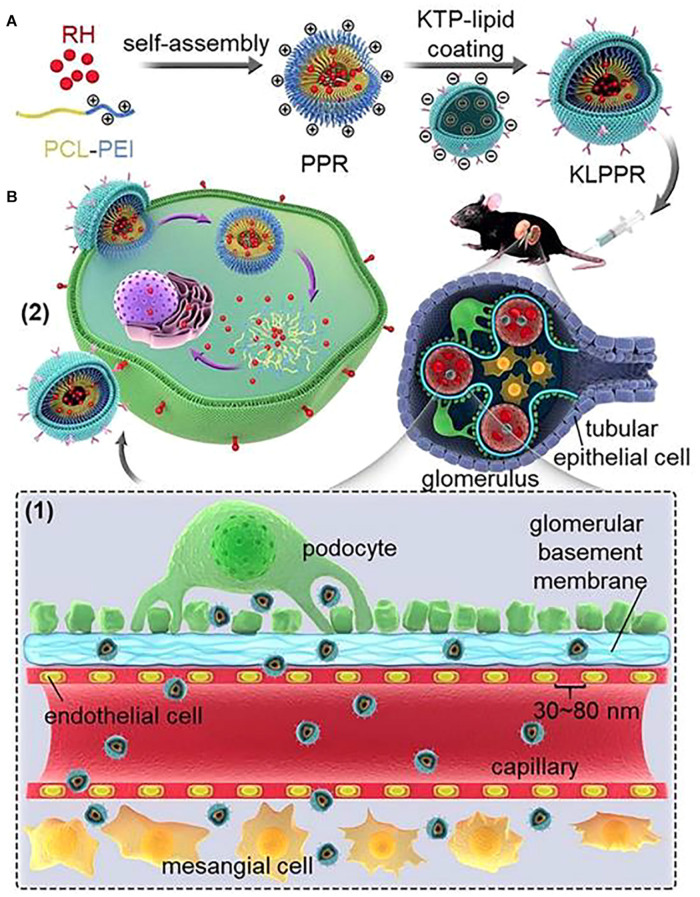
KLPPR liponanoparticles for kidney-targeted drug delivery. **(A)** Kidney-targeted rhein (RH)-loaded liponanoparticles (KLPPR) with polyethyleneimine (PCL-PEI)-based cores and KTP-modified lipid layers. **(B)** Size control (30–80 nm) enables the particle to pass through the filtration membrane, and KTP decoration promotes cellular uptake and kidney retention of the particle. Reproduced with permission (Kvirkvelia et al., 2018).

The main approach to achieve GBM and GEC targeting is peptide and antibody ligands. Wang et al. created a kidney-targeted rhein (RH)-loaded liponanoparticles (KLPPR) with polyethyleneimine-based cores and KTP-modified lipid layers. They use CLPVASC, an elastin-like polypeptide, to achieve glomerular endothelial cell barrier and basement membrane targeting (Figure 7; Wang et al., 2017). And the particle displayed excellent kidney-targeted distribution in diabetic nephropathy (Wang et al., 2019). Human monoclonal anti-α3(IV) antibody, which can specifically bind to the non-collagenous-1 domain (NC1) of α3(IV) collagen, can also be used as an antibody ligand to target drugs at glomeruli (Kvirkvelia et al., 2015, 2018).

#### Drug Delivery Systems for Podocytes

Podocytes cover the outer part of GBM, the slit between their foot processes is about 35–70 nm (Wang et al., 2017). And as podocytes are the final barrier of the glomerular filtration membrane, podocyte injury is typically related to proteinuria (Asanuma and Mundel, 2003).

##### Ultrasmall NPs

The nanocarriers have to pass through GBM (6–10 nm) before arriving at podocytes. Bruni et al. (2017) designed ultrasmall colloidal polymeric nanomaterials and try to target podocytes. The carrier is a polymer with/without a hydrophobic poly-ε-caprolactone (PCL) core and a hydrophilic PEG shell. PEG Dexamethasone was encapsulated and repairment of damaged podocytes was observed *in vitro* (Bruni et al., 2017). However, though an elevated amount of the NP in urine was observed *in vivo*, the drug accumulation is higher in the liver and the spleen than in the kidney.

##### NPs Displaying Ligands

Vascular adhesion molecule-1(VCAM-1) and cyclo-RGD peptide can be used as targeting ligands for podocytes. VCAM-1 is an inflammation-induced cell adhesion molecule, and the expression of VCAM is significantly upregulated in podocytes after stimulated by inflammatory cytokines. Visweswaran et al. (2015) constructed lipid-based nanocarriers called SAINT-O-Somes, and when displaying anti-VCAM-1 antibody ligand, the NP can target podocytes *in vitro*. *In vitro* distribution of the drug is not investigated in this study. The cyclo-RGD peptide is an αvβ3 integrin-specific ligand. αvβ3 integrin is significantly higher in diseased nephrons. Poly(N-2hydroxypropyl) methacrylamide (PHMAM), PCL and liposome-based NPs with cyclo-RGD peptide was designed (Pollinger et al., 2012; Colombo et al., 2017). The NPs can bind to the αvβ3 integrin receptor on podocytes *in vitro*. *In vivo* study in unilateral ureteral obstruction mice showed increased accumulation of RGD liposome in the fibrotic kidney (Zhou et al., 2021). When loaded with celastrol, inflammatory responses were ameliorated in glomeruli, while no obvious damages to other organs were observed.

In fact, upregulated expression of αvβ3 integrin or VCAM-1 is a common feature in various organs undergoing fibrosis, and it may not able to target specific cell types *in vivo*. But it provided a new approach to target diseased sites during kidney diseases.

#### Drug Delivery Systems for Mesangial Cells

Mesangial cells (MC) account for about 40% of glomerulus cells and are the main cellular component of mesangium. Mesangial cell injury is involved in various forms of glomerular diseases including immune-mediated glomerular diseases and metabolic diseases (Abboud, 2012). Especially, drugs including immunosuppressants targeting MCs may have great potential in improving the treatment of mesangial proliferative glomerulonephritis. To reach MCs, the NPs have to pass the glomerular endothelial barrier, whose fenestrae pore is sized 70–130 nm (Liu et al., 2019).

Theoretically, NPs sized less than this size can penetrate the pores and reach the mesangium. The size-associated accumulation is proved by PEGylated gold NPs. PEGylated gold NPs with diameters less than 100 nm can accumulate at the glomerular mesangium (Choi et al., 2011; Kamaly et al., 2016). The particles mainly accumulate at liver, spleen, and kidney, and the accumulation in liver and spleen was correlated with particle size. At the size of 75 ± 25 nm, maximum accumulation in kidney mesangium was reported.

##### PLGA-PEG NPs

Based on size selection, researchers constructed a 90 nm PLGA-PEG NPs loaded with dexamethasone acetate, the NPs can deposit at MCs and the accumulation was achieved mainly through clathrin-dependent endocytosis (Li et al., 2019). By using the NPS, dexamethasone acetate is mainly distributed in the kidney. Its accumulation in the liver is significantly reduced comparing to traditional dexamethasone acetate solution and thus can reduce systemic side effects of the drug.

##### Albumin Nanoparticles

Albumin nanoparticles (ANs) produced by human serum albumin can be used as drug carriers for mesangial cells. They are biocompatible and biodegradable. Comparing with ANs of 75 or 130 nm size, ANs of 95 nm size showed better glomerular accumulation. The reason may lie in the mechanism of cellular internalization. Researchers found that cellular uptake of ANs is energy-dependent, and macropinocytosis, caveolae- and clathrin-mediated endocytosis participate in the process. All the three pathways mentioned contribute to the internalization of ANs sized 95 nm by the mesangial cells, while only one or two pathways are involved in the uptake of ANs sized 75 and 130 nm. ANs loaded with celastrol (CLT-AN) are prominent for the treatment of mesangioproliferative glomerulonephritis. Compared to traditional celastrol treatment, celastrol encapsulated in ANs showed improved ability to ameliorate renal injuries and reduced toxicity to brain, heart, and liver in animal models (Guo et al., 2017).

##### NPs Displaying Ligands

NPs coated with targeting antibodies are also proved to be able to target at MCs in animal models: anti-integrinα8 monoclonal antibodies for normal mice and mice with lupus glomerulonephritis (Scindia et al., 2008; Liu et al., 2019) and anti-Thy-1-membrane glycoprotein for IgA nephropathy in rats (Suana et al., 2011).

##### Liposome

Wang et al. (2020) designed a PEG-modified cationic liposome with octa-arginine (R8) coating and sized about 110 nm. The liposomal nanoparticles are loaded with both p38α MAPK and p65 siRNA, and they mainly accumulate at kidney with the highest uptake by MCs in mice models (Wang et al., 2020).

##### Ultrasmall NPs

A 2016 study showed that after intravenously injected, anionic ultrasmall quantum dot NPs (∼3.7 nm) can be gradually taken up by MCs up to 30 days (Liang et al., 2016), suggesting a possible way to design MC targeting ultrasmall NPs.

## The Clearance of Kidney-Specific Delivery Nanomaterials

Though many mechanisms may be involve in the clearance of kidney-targeted NPs, there are two main ways: directly filtered by the glomeruli and excreted into the urine, or eliminated through biodegradation.

Generally, NPs with a hydrodynamic diameter smaller than 5.5 nm can pass through the glomerular filtration membrane and excrete into the urine, and NPs larger than 6 nm can be cleared by the reticuloendothelial system (Choi et al., 2007; Alexis et al., 2008; Sadauskas et al., 2009; Tsoi et al., 2016; Du et al., 2017; Poon et al., 2020). However, studies showed that some quantum dot NPs with hydrodynamic diameters smaller than 4.5 nm may also accumulate at the kidney over 15–80 days (Su et al., 2011), and positively charged NPs can be cleared from the kidney faster than those with neutral or negative surface charge (Blanco et al., 2015). A study further analyzed the fate of ultrasmall meracaposuccinic acid capped quantum dots (MSA-QDs) with negative surface charge and PEI conjugated quantum dots (PEI-QDs) (Liang et al., 2016). At 30 min after administration, positively charged NPs (PEI-QDs) were filtered into urine while negatively charged NPs (MSA-QDs) were hardly observed in the urine. And the MSA-QDs are found gradually accumulated in mesangial cells for more than 30 days.

And in another study, the clearance of a cationic, cyclodextrin-containing polymer (CDP)-based carrier was studied (Zuckerman et al., 2012). When loaded siRNA was fluorescently labeled with Cy3, the researchers found that after i.v. administration, the fluorescence signal was observed in glomerular cells within 10 min, and then decreased markedly at 15 min, and then ends up in the urine. However, a further study found that the NPs were disassembled at the GBM. Though siRNA was excreted into the urine, the NP carriers were deposited at the GBM. The researchers also found that some larger NPs also deposit at the endothelial cells of peri-tubule capillaries. The deposition pattern is also observed in pegylated gold NPs (Choi et al., 2011), so it is speculated as a generalized phenomenon for NP systems (Zuckerman et al., 2012), and further studies are needed to understand the long-term fate and toxicity of non-biodegradable kidney targeted NPs (Poon et al., 2019).

## Limitations and Future Perspectives

Currently, many hybrids especially NP-based kidney-targeted drug delivery systems have been investigated. Though most of them are at an early stage, some of them achieved kidney-specific delivery, and comparing to traditional treatment method they showed markable therapeutic effect and significantly reduced side effect when loaded with drugs in diseased animal models. Still, work should be done to translate these achievements into clinical situations, and several limitations should be overcome. The first one is biosafety. As mentioned, the long-term fate and toxicity of non-biodegradable kidney-targeted NPs are unclear, and more detailed investigations should be done before clinical use. The second one is clinical effectiveness. Most research data are acquired from animals or even cells, and there is a huge gap between animal models (or cells) and actual patients. For example, some NPs displaying ligands or antibodies that depend on inflammatory responses in the kidney may face off-target effects in patients with systemic inflammation. The third one is stableness in the manufacturing process. Producing a large scale of stable high-quality NPs is still difficult.

Biodegradable, non-toxic, non-immunogenic materials may prompt the translation from laboratory to clinical use. FDA has approved PLGA-PEG for human use. Hopefully, with the joint effort of nephrologists and technologists, these materials will provide novel approaches to kidney disease and finally apply them clinically.

## Author Contributions

WL, FH, and YL were responsible for the conception and design of the review, and revised the manuscript. XH and YM drafted the manuscript. All authors contributed to the article and approved the submitted version.

## Conflict of Interest

The authors declare that the research was conducted in the absence of any commercial or financial relationships that could be construed as a potential conflict of interest.

## Footnotes

1 https://nccd.cdc.gov/CKD
